# Comment on “Simulation-Based Mastery Learning with Deliberate Practice Improves Clinical Performance in Spinal Anesthesia”

**DOI:** 10.1155/2014/579273

**Published:** 2014-12-15

**Authors:** Kieran Walsh

**Affiliations:** BMJ Learning, BMA House, Tavistock Square, London WC1H 9JR, UK

Udani et al. have presented the results of a straightforward study on the effect of simulation-based mastery learning with deliberate practice on clinical performance in spinal anesthesia [1]. And the results of the study are equally straightforward: simulation-based mastery learning with deliberate practice appears to improve the skills of learners.

However, there are a number of reasons to be concerned about these results. First of all, one half of the group received deliberate practice plus the base curriculum, but the other half received only the base curriculum. It is perhaps unsurprising that a group that receives more high quality education will achieve better outcomes than a group that receives less. The extent to which these results further the evidence base is therefore questionable. Perhaps if both groups received a different form of deliberate practice and one group achieved better outcomes, we would know more about the forms of deliberate practice that are most effective. However, this is not the study that was performed. There is now a growing body of evidence as to the effectiveness of deliberate practice and simulation-based mastery learning as a pedagogical method. The state of the science is now such that quite soon further studies on the effectiveness of deliberate practice will be redundant. At that stage it will be even more important to look into the “black box” of this medical education intervention to find out how it works and what aspects of it are particularly effective.

Secondly, the group that did not receive the deliberate practice might have reason to feel that they were shortchanged in the study. They simply did not receive all the education that they might have done. In fairness to the authors, the institutional review board approved the study and the learners consented to taking part, so the research was undertaken with high levels of transparency. Perhaps all learners were given the opportunity to undertake deliberate practice once the study was completed, in which case all would have felt that they were treated equally.

On a final note, the authors state that a potential limitation was the low number of study participants. But they are correct that it is difficult in practical terms to do studies with large numbers of participants. From another perspective, however, the small size adds credence to their findings. To be able to demonstrate positive results with a small sample size implies that the improvement is real and not just statistically significant but educationally significant as well.
